# Recent Advances and Future Challenges in the Genetics of Multiple Sclerosis

**DOI:** 10.3389/fneur.2014.00130

**Published:** 2014-07-14

**Authors:** Christina M. Lill

**Affiliations:** ^1^Neuropsychiatric Genetics Group, Department of Vertebrate Genomics, Max Planck Institute for Molecular Genetics, Berlin, Germany; ^2^Focus Program Translational Neuroscience, Department of Neurology, University Medical Center of the Johannes Gutenberg University Mainz, Mainz, Germany

**Keywords:** immunogenetics, autoimmune disease, multiple sclerosis, genome-wide association study, epigenetics, rare variants, heritability

## Abstract

Multiple sclerosis (MS) is the most common auto-inflammatory disease of the central nervous system, affecting more than 2 million individuals worldwide. It is a genetically complex disease, in which a substantial part of a person’s liability to develop MS is caused by a combination of multiple genetic and non-genetic (e.g., environmental) risk factors. Increasing this complexity, many of the involved risk factors likely interact in an intricate and hitherto ill-defined fashion. Despite these complexities, and owing greatly to the advent and application of large-scale genome-wide association studies, our understanding of the genetic factors underlying MS etiology has begun to gain unprecedented momentum. In this perspective, I will summarize some recent advances and outline future challenges in MS genetics research.

## Introduction: The “Heritable” Basis of Multiple Sclerosis

It was already recognized decades ago that multiple sclerosis (MS) aggregates within families – ~20% of all patients of European descent show a positive family history in comparison to a general MS prevalence of 0.5–0.1% (1). Population-based family studies including the investigation of mono- vs. dizygotic twin pairs revealed that the observed familial aggregation is likely due to shared heritable factors, while the influence of shared environmental factors on family aggregation is comparatively small. Rather, environmental factors are believed to act on a non-shared, population-wide basis (2). Previously reported estimates of heritability, i.e., the proportion of phenotypic variance attributable to heritable factors, range from 25 to 76%. This is consistent with the estimate of the largest population-based study to date based on a Swedish dataset published earlier this year (64%, confidence interval 36–76%) (2). However, in contrast to other neurologic disorders such as Alzheimer’s (AD) or Parkinson’s disease (PD), there is little evidence that fully penetrant, causative mutations in single genes account for the family aggregation observed in MS. Instead, MS heritability appears to be exclusively governed by hundreds to thousands of common genetic variants [e.g., single-nucleotide polymorphisms (SNPs) with minor allele frequencies (MAF) ≥0.5%] exerting small to moderate risk effects (3). High-throughput genotyping studies are the key instruments to identify such disease-associated “polymorphisms,” e.g., by comparing a variant’s allele frequency in a group of unrelated MS cases and controls.

## Recent Advances in MS Genetics Research

The major histocompatibility complex (MHC) region on chromosome 6p21.32 is the first identified MS risk locus from the candidate-gene era [originally detected over 40 years ago (4, 5)] that is still valid today. As a matter of fact, with the class II HLA-DRB1*1501 allele conferring a ~3-fold risk increase [as measured by the odds ratio (OR)], the MHC region still represents the most important MS risk locus by far (6). Following the discovery of MHC in MS, literally hundreds of candidate-gene based association studies investigating hundreds of genes were published with contradictory results. Among these, IL7RA (encoding the interleukin receptor 7A) was initially assessed and reported as a putative MS risk gene using a candidate-gene approach (7–9) and still shows convincing evidence for association with MS today (6, 10). However, it was only since the first applications of the genome-wide association study (GWAS) approach that the majority of today’s established genetic association findings were uncovered: since 2007 several MS GWAS and follow-up studies have been published each expanding the number of (mostly) genuine risk loci. As in other complex diseases (11), the continuously increasing size of the study samples along with the application of stringent *p* value thresholds (i.e., the genome-wide significant threshold, see Glossary) was the most important contributor to the growing number of replicable risk SNPs.

The most recent and largest GWAS (6) to date analyzed 465,434 SNPs across ~9,800 cases and 17,400 controls. The most interesting results were subsequently followed-up in ~11,500 additional subjects. This seminal study (6) not only confirmed 23 MS risk loci reported by previous GWAS, but also identified 29 novel and genome-wide significant signals outside the MHC region. In addition, several loci showed suggestive (i.e., sub genome-wide) significant evidence for association with MS, 5 of which were subsequently confirmed to be genuine MS risk loci after testing another ~20,000 independent cases and controls (12). Finally, conditional analyses of variants within the MHC locus revealed at least three additional susceptibility loci that are associated with MS risk independent of the HLA-DRB1*1501 allele: while the class I allele HLA-A*0201 showed additional protective effects, class II alleles DRB1*0301 (or DQB1*0201, since the signals could not be separated) as well as DRB1*1303 conferred further risk effects (6). Additional large-scale association studies (10, 13) – including a recent follow-up project in ~14,500 independent MS cases and 24,100 controls using the “ImmunoChip” genotyping array [customized for targeting several hundred loci previously implicated in autoimmune diseases (10)] – substantially extended the list of genome-wide significant non-MHC MS risk loci to 103. These non-MHC risk loci established by GWAS and large-scale follow-up studies show moderate to modest effects with OR of ~1.05 to 1.30.

Gene-ontology analyses based on these established MS risk loci have confirmed their role in T cell mediated immunity while a primary neurodegenerative role – a hypothesis discussed not too long ago – appears to be negligible (6, 10). Along these lines, MS risk genes substantially overlap with GWAS findings from other autoimmune diseases (6) but not with those reported for neurodegenerative diseases (14). Interestingly, the list of established risk loci discovered in autoimmune diseases such as MS, Crohn’s disease (CD), and type I diabetes (T1D) substantially outnumbers those identified in primarily neurodegenerative diseases such as AD or PD (>50 vs. <20 per disease trait) despite comparable study designs and sample sizes (11). The reasons for this stark difference remain unclear at this time.

## Searching for the “Missing Heritability”

Despite the impressive number of common MS risk variants identified over the years, the proportion of heritability they explain remains modest owing to their small effect sizes. Similar observations have been made in many other genetically complex diseases. Some authors have thus coined the term of “missing heritability” in complex diseases (15). Recent estimates suggest that the MS risk loci identified to date explain only about one-quarter (~27%) of its total heritability most of which is attributable to the MHC locus (10).

Undoubtedly, the next round of even larger-scale efforts such as “mega” meta-analyses of all independent GWAS datasets and follow-up studies will identify additional genome-wide significant MS risk loci. Unless they were previously “missed” due to technical reasons (e.g., insufficient capture by GWAS microarrays), these additional genetic risk loci will either consist of frequent variants (with MAF ≥ 5%) that exert small to very small effects (ORs < 1.10), or less frequent variants (MAF <5 and ≥0.5%) exerting slightly stronger effects (ORs >1.2 and <2). In either setting, the identified loci would only moderately improve the proportion of heritability explained. However, a substantial fraction of heritability may be accounted for by a multitude of genetic variants that may never surpass the genome-wide significance threshold in association studies due to very small effect sizes as demonstrated previously (16).

Another popular hypothesis posits that a sizeable fraction of the hitherto missing heritability in MS (and other diseases) may be explained by the presence of rare variants (i.e., those with a MAF <0.5% in the general population) exerting much larger effects than common variants (ORs (2). Due to their low frequency they are often not accurately captured by current GWAS arrays. However, in disagreement with this “rare variant hypothesis” a recent proof-of-principle study applying next-generation re-sequencing of 25 previously reported autoimmune GWAS loci (including 10 MS loci) found no evidence for the existence of rare high-risk variants in autoimmune diseases in any of the tested regions (17). As GWAS loci presumably have a higher probability of harboring rare, deleterious alleles than the rest of the genome, the authors concluded that rare variants unlikely account for a substantial fraction of missing heritability in autoimmune diseases (17). Along these lines, an initial report (18) of a rare, high-risk MS variant in the vitamin-D activating gene CYP27B1 was not confirmed in independent validation studies (19, 20). Future genome-wide studies need to test massive sample sizes, comparable with those used in recent GWAS, to conclusively assess the potential role of rare variants in MS. Still, even if a number of genuine rare variants increasing MS risk were uncovered, these would only explain a very small proportion of the missing heritability unless they should exist in very large numbers, which is unlikely.

An alternative hideout for the missing heritability in MS may lie behind structural genomic variations, e.g., copy number variants (CNVs). While these have not been systematically assessed on a genome-wide level in MS yet, a study led by the Welcome Trust Case Control Consortium assessed common CNVs across eight diseases including CD, rheumatoid arthritis (RA), and T1D, but only three association signals emerged from this analysis (two for CD and one for T1D), all of which were also tagged by SNPs (21). In light of these findings, CNVs may not represent compelling candidates to explaining a major proportion of missing MS heritability.

This leaves heritable epigenetic variations, such as DNA methylation, histone modifications, and inherited expression of non-coding RNAs to be reasonable culprits in the quest for the missing heritability in MS. Within recent years, technologies that allow the assessment of DNA methylation on a genome-wide level have become affordable for large sample sizes. However, evidence in favor of transgenerational epigenetic effects in genetically complex diseases is currently limited to results in animal models and only very few epidemiological studies (22, 23). In MS, these latter studies do not show convincing evidence of a parent-of-origin effect, which could be supportive for the presence of major transgenerational epigenetic risk factors. Specifically, results from earlier studies suggesting maternal parent-of-origin effects in MS [e.g., Ref. (24, 25)] were not validated by a much larger epidemiological study (2). As a matter of fact, this latter study indicated modest evidence for a paternal parent-of-origin effect, which would be compatible with a modest Carter effect (2). Notwithstanding, while the absence of conclusive evidence for parent-of-origin effects may exclude major contributions of few epigenetic factors to MS heritability, it does not preclude the action of multiple heritable epigenetic factors of small effect, which can be effective in germ cells of both parents. Unfortunately, the experimental exploration of this hypothesis is difficult, as epigenetic marks often differ in a tissue-specific manner and may change over time, complicating the design and interpretation of epigenetic association studies (26).

In addition to the above considerations, there are other aspects to keep in mind when trying to fill the missing heritability gap. One is the clinical and paraclinical heterogeneity of MS that may at least in part reflect differences in its genetic architecture (27). Examples of such differences have been reported for a number of other genetically complex diseases (27). However, data to support the presence of differing genetic risk profiles in MS remain limited: recent studies have suggested that primary progressive (PP) and bout onset MS forms do not differ in their spectrum of genetic risk factors (6, 28); however, these results are based on limited numbers of patients (owing to the PP prevalence of 10–20% among MS cases) and may not represent definite answers. Furthermore, previous genome-wide efforts have reported only sub-genome-wide significant results for association with MS severity (6, 29, 30) or for effect size differences in HLA-DRB1*1501 carriers and non-carriers. Analyses of age of onset yielded only one genome-wide significant association signal, which corresponds to the HLA-DRB1*1501 allele (6). The failure to identify convincing associations in most subgroup analyses may partly be due to limitations in power in some studies, potential issues with misclassification with regard to clinical variables, and, as suggested recently, the use of suboptimal quantification systems of clinical parameters (31). Thus, genome-wide risk analyses of more appropriately classified MS subgroups and/or other clinical and paraclinical variables may shed new light on potential risk factors for certain MS endophenotypes.

Last but not least, our estimates of the proportion of explained heritability are based on the combined additive effects of all known risk loci (weighted by their frequencies) relative to the total heritability estimated in populations (e.g., based on family studies, see above). However, this population-derived total heritability estimate very likely consists of additive effects of single loci as well as – quite substantially, as argued by some authors – effects due to gene–gene (GxG, a.k.a. epistatic) and gene–environment (GxE) interactions (10, 32). Unfortunately, there is currently no robust knowledge about GxG and GxE interaction effects in MS (and, as a matter of fact, for most other diseases). Sufficiently powered GxG interaction studies on a genome-wide basis require unrealistically large sample sizes (i.e., ≥450,000 subjects) (32), making it unlikely that the near future will yield substantial progress in this field. Likewise, genome-wide GxE analyses have not been performed for MS, either. They experience the difficulties of collecting both large, preferably population-based DNA samples and relevant high quality environmental data for a disease with comparatively low prevalence.

## Unveiling the Etiology of MS Using Genetics

As outlined above, the individual and collective contribution of the hitherto identified non-MHC genetic risk variants to MS risk is modest at best, and individual risk prediction is not possible based on the current knowledge. As a result, one could argue that this line of research has only negligibly impacted our understanding of MS pathophysiology and bears little medical relevance. However, this conclusion would be premature, especially given the short time frame that the GWAS methodology has been applied to the field of MS genetics. By design, SNPs assessed in GWAS can be informative markers tagging chromosomal regions associated with disease, but often do not represent the actual variants exerting a direct functional effect, some associated SNPs may even be located between genes. Thus, the identification of the functional variants and the elucidation of underlying pathomechanisms will pose a major challenge in MS genetics research in the coming years. One recent example providing novel and intriguing insights into MS pathophysiology is the successful functional characterization of the MS risk variant underlying the association signal (6) on chromosome 12p13.31 (33). This region contains the gene TNFRSF1A, which encodes the tumor necrosis factor (TNF) receptor superfamily member 1A. Binding of TNF to the extracellular domain of the membrane-bound TNF receptor 1A initiates activation of the NF-κB pathway and apoptosis of immune cells. Genetic re-analysis (“fine-mapping”) of the MS GWAS data revealed that the originally identified MS-associated intronic SNP (6), rs1800693, is also likely to be the functional SNP (33). Subsequent functional experiments showed that this variant leads to alternative splicing, resulting in a shortened soluble TNFRSF1A isoform that lacks apoptotic activity but binds and thus neutralizes TNF (33). Intriguingly, anti-TNFα therapy induces onset and exacerbation of MS, thus mimicking the effect of the MS risk variant (33). Furthermore, this anti-TNFα therapy is successful in treating other autoimmune diseases such as RA and CD, and in these diseases, rs1800693 or other polymorphisms in TNFRSF1A do not show evidence for genetic association (11, 33).

## Conclusion

The TNFRSF1A example clearly demonstrates that systematic follow-up of specific variants showing convincing association with MS risk, regardless of the underlying effect size, can reveal valuable insights into the disease’s etiology and even pinpoint novel therapeutic strategies. Thus, while we may never be able to entirely explain MS heritability by means of genetic association analyses, progress in this field of research can be expected to dramatically increase our understanding of the underlying pathophysiology and to inform the development of novel biomarkers and improved treatment strategies.

## Glossary

*Candidate-gene study*: a study that assesses the association between a limited number of genetic variants in genes with a plausible role in disease pathophysiology and a phenotype of interest, e.g., disease status.

*Copy number variant*: structural variation in the genome with an abnormal number of DNA sequence stretches (spanning several hundreds to a few million bases of DNA), resulting in deletions or multiplications (e.g., duplications).

*Epigenetics*: the study of mechanisms that lead to alterations of gene expression that are not due to changes of the primary DNA sequence; examples include DNA methylation, histone modification, and the involvement of non-coding RNAs.

*Genome-wide association study (GWAS)*: a study that assesses the association between several hundred thousands to millions of polymorphisms across the entire genome and a phenotype of interest based on data from one single experiment using specific GWAS microarrays.

*Genome-wide significance*: the significance threshold (most commonly used: *p* = 5 × 10^−8^) that is required to declare the presence of genetic association in the context of genome-wide analyses as well as in targeted genetic association studies.

*Heritability*: the proportion of phenotypic variation, e.g., case/control status that is due to heritable factors. Heritability is usually estimated in population-based family studies, e.g., twin studies.

*Next-generation sequencing*: ultra high-throughput sequencing technology that can generate millions of DNA sequence reads in one experiment; the very high efficiency of this technology is achieved by massive parallelization of sequencing reactions.

*Single-nucleotide polymorphism (SNP)*: a single base pair variant in the genome that is relatively common in the general population based on arbitrary frequency cut-offs for the minor allele (e.g., 0.5%).

## Conflict of Interest Statement

The author declares that the research was conducted in the absence of any commercial or financial relationships that could be construed as a potential conflict of interest.

## References

[1] Compston A Coles A Multiple sclerosis. Lancet (2008) 372:1502–17 10.1016/S0140-6736(08)61620-7 18970977

[2] Westerlind H Ramanujam R Uvehag D Kuja-Halkola R Boman M Bottai M Modest familial risks for multiple sclerosis: a registry-based study of the population of Sweden. Brain (2014) 137:770–8 10.1093/brain/awt356 24441172 PMC3927700

[3] International Multiple Sclerosis Genetics Consortium (IMSGC) Bush WS Sawcer SJ de Jager PL Oksenberg JR McCauley JL Evidence for polygenic susceptibility to multiple sclerosis – the shape of things to come. Am J Hum Genet (2010) 86:621–5 10.1016/j.ajhg.2010.02.027 20362272 PMC2850422

[4] Bertrams J Kuwert E HL-A antigen frequencies in multiple sclerosis. Significant increase of HL-A3, HL-A10 and W5, and decrease of HL-A12. Eur Neurol (1972) 7:74–8 10.1159/000114414 5019156

[5] Naito S Namerow N Mickey MR Terasaki PI Multiple sclerosis: association with HL-A3. Tissue Antigens (1972) 2:1–4 10.1111/j.1399-0039.1972.tb00111.x 5077731

[6] International Multiple Sclerosis Genetics Consortium, Wellcome Trust Case Control Consortium 2 Sawcer S Hellenthal G Pirinen M Spencer CC Genetic risk and a primary role for cell-mediated immune mechanisms in multiple sclerosis. Nature (2011) 476:214–9 10.1038/nature10251 21833088 PMC3182531

[7] Teutsch SM Booth DR Bennetts BH Heard RNS Stewart GJ Identification of 11 novel and common single nucleotide polymorphisms in the interleukin-7 receptor-alpha gene and their associations with multiple sclerosis. Eur J Hum Genet (2003) 11:509–15 10.1038/sj.ejhg.5200994 12825072

[8] Zhang Z Duvefelt K Svensson F Masterman T Jonasdottir G Salter H Two genes encoding immune-regulatory molecules (LAG3 and IL7R) confer susceptibility to multiple sclerosis. Genes Immun (2005) 6:145–52 10.1038/sj.gene.6364171 15674389

[9] Gregory SG Schmidt S Seth P Oksenberg JR Hart J Prokop A Interleukin 7 receptor alpha chain (IL7R) shows allelic and functional association with multiple sclerosis. Nat Genet (2007) 39:1083–91 10.1038/ng2103 17660817

[10] International Multiple Sclerosis Genetics Consortium (IMSGC) Beecham AH Patsopoulos NA Xifara DK Davis MF Kemppinen A Analysis of immune-related loci identifies 48 new susceptibility variants for multiple sclerosis. Nat Genet (2013) 45:1353–60 10.1038/ng.2770 24076602 PMC3832895

[11] Welter D MacArthur J Morales J Burdett T Hall P Junkins H The NHGRI GWAS Catalog, a curated resource of SNP-trait associations. Nucleic Acids Res (2014) 42:D1001–6 10.1093/nar/gkt1229 24316577 PMC3965119

[12] International Multiple Sclerosis Genetics Consortium Lill CM Schjeide BM Graetz C Ban M Alcina A MANBA, CXCR5, SOX8, RPS6KB1 and ZBTB46 are genetic risk loci for multiple sclerosis. Brain (2013) 136:1778–82 10.1093/brain/awt101 23739915 PMC3673463

[13] Lill CM Schjeide BM Graetz C Liu T Damotte V Akkad DA Genome-wide significant association of ANKRD55 rs6859219 and multiple sclerosis risk. J Med Genet (2013) 50:140–3 10.1136/jmedgenet-2012-101411 23315543

[14] Lill CM Bertram L Towards unveiling the genetics of neurodegenerative diseases. Semin Neurol (2011) 31:531–41 10.1055/s-0031-1299791 22266890

[15] Manolio TA Collins FS Cox NJ Goldstein DB Hindorff LA Hunter DJ Finding the missing heritability of complex diseases. Nature (2009) 461:747–53 10.1038/nature08494 19812666 PMC2831613

[16] Yang J Benyamin B McEvoy BP Gordon S Henders AK Nyholt DR Common SNPs explain a large proportion of the heritability for human height. Nat Genet (2010) 42:565–9 10.1038/ng.608 20562875 PMC3232052

[17] Hunt KA Mistry V Bockett NA Ahmad T Ban M Barker JN Negligible impact of rare autoimmune-locus coding-region variants on missing heritability. Nature (2013) 498:232–5 10.1038/nature12170 23698362 PMC3736321

[18] Ramagopalan SV Dyment DA Cader MZ Morrison KM Disanto G Morahan JM Rare variants in the CYP27B1 gene are associated with multiple sclerosis. Ann Neurol (2011) 70:881–6 10.1002/ana.22678 22190362

[19] Ban M Caillier S Mero IL Myhr KM Celius EG Aarseth J No evidence of association between mutant alleles of the CYP27B1 gene and multiple sclerosis. Ann Neurol (2013) 73:430–2 10.1002/ana.23833 23444327 PMC3631291

[20] Barizzone N Pauwels I Luciano B Franckaert D Guerini FR Cosemans L No evidence for a role of rare CYP27B1 functional variations in multiple sclerosis. Ann Neurol (2013) 73:433–7 10.1002/ana.23834 23483640

[21] Wellcome Trust Case Control Consortium Craddock N Hurles ME Cardin N Pearson RD Plagnol V Genome-wide association study of CNVs in 16,000 cases of eight common diseases and 3,000 shared controls. Nature (2010) 464:713–20 10.1038/nature08979 20360734 PMC2892339

[22] Petronis A Epigenetics as a unifying principle in the aetiology of complex traits and diseases. Nature (2010) 465:721–7 10.1038/nature09230 20535201

[23] Grossniklaus U Kelly WG Ferguson-Smith AC Pembrey M Lindquist S Transgenerational epigenetic inheritance: how important is it? Nat Rev Genet (2013) 14:228–35 10.1038/nrg3435 23416892 PMC4066847

[24] Ebers GC Sadovnick AD Dyment DA Yee IM Willer CJ Risch N Parent-of-origin effect in multiple sclerosis: observations in half-siblings. Lancet (2004) 363:1773–4 10.1016/S0140-6736(04)16304-6 15172777

[25] Hoppenbrouwers IA Liu F Aulchenko YS Ebers GC Oostra BA van Duijn CM Maternal transmission of multiple sclerosis in a Dutch population. Arch Neurol (2008) 65:345–8 10.1001/archneurol.2007.63 18332246

[26] Rakyan VK Down TA Balding DJ Beck S Epigenome-wide association studies for common human diseases. Nat Rev Genet (2011) 12:529–41 10.1038/nrg3000 21747404 PMC3508712

[27] Ramanujam R Ramanujam S Hillert J Considerations for subgroups and phenocopies in complex disease genetics. PLoS One (2013) 8:e71614 10.1371/journal.pone.0071614 23977088 PMC3748062

[28] Isobe N Damotte V Lo Re V Ban M Pappas D Guillot-Noel L Genetic burden in multiple sclerosis families. Genes Immun (2013) 14:434–40 10.1038/gene.2013.37 23903824 PMC4102601

[29] Brynedal B Wojcik J Esposito F Debailleul V Yaouanq J Martinelli-Boneschi F MGAT5 alters the severity of multiple sclerosis. J Neuroimmunol (2010) 220:120–4 10.1016/j.jneuroim.2010.01.003 20117844

[30] International Multiple Sclerosis Genetics Consortium. Genome-wide association study of severity in multiple sclerosis. Genes Immun (2011) 12:615–25 10.1038/gene.2011.34 21654844 PMC3640650

[31] Sawcer S Franklin RJM Ban M Multiple sclerosis genetics. Lancet Neurol (2014) 13(7):700–9 10.1016/S1474-4422(14)70041-9 24852507

[32] Zuk O Hechter E Sunyaev SR Lander ES The mystery of missing heritability: genetic interactions create phantom heritability. Proc Natl Acad Sci U S A (2012) 109:1193–8 10.1073/pnas.1119675109 22223662 PMC3268279

[33] Gregory AP Dendrou CA Attfield KE Haghikia A Xifara DK Butter F TNF receptor 1 genetic risk mirrors outcome of anti-TNF therapy in multiple sclerosis. Nature (2012) 488:508–11 10.1038/nature11307 22801493 PMC4268493

